# Deep Multi-Scale Residual Connected Neural Network Model for Intelligent Athlete Balance Control Ability Evaluation

**DOI:** 10.1155/2022/9012709

**Published:** 2022-05-26

**Authors:** Nannan Xu, Xin Wang, Yangming Xu, Tianyu Zhao, Xiang Li

**Affiliations:** ^1^Sports Training Institute, Shenyang Sport University, Shenyang 110115, China; ^2^Department of Kinesiology, Shenyang Sport University, Shenyang 110115, China; ^3^School of Mechanical Engineering and Automation, Northeastern University, Shenyang 110819, China; ^4^Key Laboratory of Structural Dynamics of Liaoning Province, College of Sciences, Northeastern University, Shenyang 110819, China; ^5^Key Laboratory of Education Ministry for Modern Design and Rotor-Bearing System, Xi'an Jiaotong University, Xi'an 710049, China

## Abstract

Athlete balance control ability plays an important role in different types of sports. Accurate and efficient evaluations of the balance control abilities can significantly improve the athlete management performance. With the rapid development of the athlete training field, intelligent and automatic evaluations have been highly demanded in the past years. This study proposes a deep learning-based athlete balance control ability evaluation method through processing the time-series movement pressure measurement data. An end-to-end model structure is proposed, which directly analyzes the raw data and provides the evaluation results, which largely facilitates practical utilization. A multi-scale feature extraction scheme is employed, by exploring the learned features in different scales. A residual connected neural network architecture is further proposed. By using the short-cut connection, the deep neural network model can be more efficiently trained. Experiments on the real athlete balance control ability tests are carried out for validations. Through comparisons with different related methods, the results show the proposed deep multi-scale residual connected neural network model is well suited for the athlete balance control ability evaluation problem, and promising for actual applications in the real scenarios.

## 1. Introduction

Balance control ability is of great importance for athletes. A number of sport areas with precise movement require accurate and efficient evaluations of the balance control ability for the athletes, such as freestyle skiing aerials, skating, and so forth [1]. Good evaluations of the balance control ability can well support the management of the athletes, including selection, training, competition, and so on. Accurate evaluation of the balance control ability remains a quite challenging issue, since a large number of factors are included, and the underlying ability cannot be well reflected. Significant expert knowledge and human labor are also highly required for this task, which makes it difficult to be carried out in the practical scenarios [2].

In the recent years, with the rapid development of the sensing technologies and data analysis methods, data-driven athlete balance control ability evaluation becomes feasible [3]. Specifically, the movement pressure measurement machine, such as a balance meter, can be used to collect the athlete subtle movement when they are standing on the machine. The collected signal can be used to evaluate the athlete balance control ability, since smaller movement pressure generally indicates better balance control ability, while larger movement pressure means the balance control ability is at lower level [4].

With respect to the collected data, typical statistical features can be used for balance control ability evaluation, such as mean, root mean square, and so forth [5–7]. In the past years, many signal processing methods have been proposed for better feature extraction [8–10], including wavelet analysis, stochastic resonance techniques, and so on. Some machine learning and statistical inference techniques are also developed for solving the pattern recognition problem, such as artificial neural networks (ANN) [11], support vector machines (SVM), random forest, fuzzy inference, and so on [12–14]. However, the collected movement pressure data usually contain much noise, which makes it difficult to use the conventional features for evaluations. Furthermore, for the high-level athletes, the difference between different levels of athlete on balance control ability is quite small. The typical features cannot well reflect the difference. Therefore, the traditional data-driven methods on athlete balance control ability evaluation are facing great challenges at present.

Deep neural network has been the emerging technologies of artificial intelligence in the past years [15–23], and it has achieved great successes in many applications such as image recognition [24–26] and speech recognition [27]. Driven by big data, deep neural network can well learn the mapping function between the input data and the output pattern automatically [28–30]. High prediction accuracy can be usually obtained. In addition, deep neural network is generally a black box tool for automatic computations, which requires little prior knowledge on signal process or domain expertise [31]. Therefore, it is quite promising for solving the challenging athlete balance control ability evaluation problem.

The recent studies [32–35] show the time-series data can be well processed by the deep neural network model, and higher feature extraction efficiency and better effects can be generally obtained using deep learning [36, 37]. Different types of time-series data have been successfully processed using deep learning, including the medical data, financial data, condition monitoring data, and so on. Miao [38] proposed a deep learning framework for continuous blood pressure measurement using one-channel ECG signal. Promising effects have been obtained for processing the pressure data. An end-to-end intelligent morphological classification method for intracranial pressure pulse waveforms was proposed in the studies [39], where the deep learning method was applied for automatic feature extraction and pattern learning.

However, the typical deep neural network model suffers from many factors. For instance, the training efficiency is generally weak with the deep architecture [10, 40–45]. Traditional model establishment approach basically loses feature information in the feed-forward manner with a single-scale feature extraction scheme. The limitations hinder the development of the deep neural network methods.

In this study, a novel deep multi-scale residual connected neural network model is proposed to address the athlete balance control ability evaluation problem, as well as the remaining problems of deep neural networks. The main novelties and contributions of this study are listed as follows:

- A new multi-scale feature extraction scheme is proposed, which consists of automatic feature learning in different scales. The integration of multi-scale features further enhances the information fusion performance and leads to better results.
- A deep residual connected module is proposed, which introduces short-cut connection between different convolutional layers in the deep neural network model. In this way, the training efficiency can be largely enhanced.
- The athlete balance control ability evaluation problem is investigated, and an intelligent method is proposed to achieve automatic feature extraction and evaluation. This has been seldomly studied in the current literature, and this study provides new insight in this task.
- Experiments on the real athlete under-feet movement pressure measurement data are used for validations of the proposed method. The results show that the proposed method can achieve high evaluation accuracy, and promising for applications in the real scenarios.

This study starts with the description of the preliminaries in Section 2. The proposed deep multi-scale residual connected method is presented in Section 3. Experiments are carried out for validations of the proposed method, and the results are shown in Section 4. We close the study with conclusions in Section 5.

## 2. Preliminaries

In this section, the preliminaries that are used in this study are presented, including the convolutional neural network, pooling, and softmax function. The concerned problem in this study can be formulated as learning a mapping function, which projects the raw collected athlete time-series data to the corresponding balance control ability level. The relationship is complex, and the traditional methods cannot well address this problem. Therefore, we propose a deep learning-based approach for modeling the highly nonlinear relationship.

### 2.1. Convolutional Neural Network

Convolutional neural networks (CNNs) have been one of the most popular neural network structures in the current literature. The effectiveness of CNNs has been widely validated in many application scenarios, such as the image classification tasks, speech recognition problems, and video processing tasks [46]. The variable and complicated signals can be automatically processed using CNNs, and high-level features can be effectively extracted. In the recent years, many researches have been carried out using CNNs and achieved significant successes [43, 47].

The most representative features of CNNs are the local receptive fields and shared parameters in signal processing. The data shift of the input data can be efficiently filtered out during feature extraction, and the spatial sub-sampling algorithm can well extract the most remarkable features from the collected data. In this study, CNNs are used as the main framework for the data-driven intelligent feature extraction of the signal.

To be specific, the convolutional layers are placed to convolve different filters with respect to the raw data, and high-level features can be obtained accordingly. In most cases, the pooling operations are used after the convolutional operations, which can further extract the most significant features for the following processing. Meanwhile, the feature dimension can be also well reduced, which benefits the processing costs.

In this study, the data are a sequence of the time-series collections. Therefore, the 1-dimensional (1D) CNN is mostly adopted for the data processing, and that will be presented in the following. Let **x**=[*x*_1_, *x*_2_,…, *x*_*N*_] denote the input data, where *N* represents the dimension of the input data sample. The convolutional computation can be defined using the filter kernel **w**, **w** ∈ *R*^*F*_*L*_^, where *F*_*L*_ represents the size of the filter kernel, defining the dimension of the local receptive field. The concatenation vector **x**_*i*:*i*+*F*_*L*_−1_ can be defined as

(1) $$\begin{array}{c} x_{i:i+F_{L}-1}=x_{i}⊕x_{i+1}⊕\cdots ⊕x_{i+F_{L}-1}, \end{array}$$

where the item *x*_*i*:*i*+*F*_*L*_−1_ is defined as the window with *F*_*L*_ sequential data points starting from the *i*-th data point. The operation ⊕ is for concatenating the concerned data into a larger information entity. At last, the convolution computation can be expressed as

(2) $$\begin{array}{c} k_{i}=\eta \left(w^{T}x_{i:i+F_{L}-1}+m\right). \end{array}$$

In this equation, *m* and *η* denote the bias vector and the neural network activation function, respectively. The feature map output *k*_*i*_ is known as the obtained features with respect to the filter kernel. Through applying the filter kernel from the first data point to the end on the input data sample, the learned feature representation can be calculated as

(3) $$\begin{array}{c} k_{j}=\left[k_{j}^{1},k_{j}^{2},\ldots ,k_{j}^{N-F_{L}+1}\right]. \end{array}$$

The expressions above represent the learned features. In the actual applications of the CNNs, a number of convolutional kernels can be used in one layer to obtain richer information from the raw data.

### 2.2. Pooling

In the typical neural networks, after the convolutional layer, a pooling layer is usually used for further feature extraction with respect to the learned feature maps. There are mainly two reasons for the utilization of pooling operations. First, the most significant features can be usually extracted by using the simple pooling functions, which provides an easy way for efficient learning. Second, the dimension of the feature maps can be largely reduced, which can help increase the processing efficiency. In this study, the max-pooling function is used, which has been popularly adopted in the literature for the related classification problems. Let *p* denote the size of the pooling opeartion. With respect to the extracted feature maps from the convolutional layers, the pooled features can be expressed as

(4) $$\begin{array}{c} q_{j}=\left[q_{j}^{1},q_{j}^{2},\ldots ,q_{j}^{s}\right],\\ q_{j}^{z}=\max\left(k_{j}^{\left(z-1\right)p+1},k_{j}^{\left(z-1\right)p+2},\ldots ,k_{j}^{\text{zp}}\right), \end{array}$$

where **q**_*j*_ represents the obtained features from the pooling operation on the *j*-th feature map that has the size of *s*.

### 2.3. Softmax Function

Softmax function is a popular function in the data-driven neural network-based classification tasks. It is usually adopted at the end layer of the deep neural network. The values of the neurons can be transformed to the predicting probabilities by using the softmax function [25]. Specifically, after multiple combinations of convolutional and pooling layers in the deep neural network, the final extracted features are the input of the softmax function. Let **x**^(*i*)^ denote the training samples, and *r*^(*i*)^ denote the corresponding class labels of the training samples. *i*=1,2,…, *N*_*tr*_, where *N*_*tr*_ represents the training data sample number. We also have **x**^(*i*)^ ∈ *R*^*N*×1^ and *r*^(*i*)^ ∈ {1,2,…, *B*}, where *B* represents the total number of concerned classes in the problem. With respect to the input data sample **x**^(*i*)^, the softmax function can well predict the class probability *p*(*r*^(*i*)^=*j| ***x**^(*i*)^),  *j*=1,2,…, *B* for different class labels. The calculated probabilities of the data samples for each class can be computed based on the hypothesis function

(5) $$\begin{array}{c} J_{\lambda }\left(x^{\left(i\right)}\right)=\left[\begin{array}{c} p\left(r^{\left(i\right)}=1|x^{\left(i\right)};\lambda \right)\\ p\left(r^{\left(i\right)}=2|x^{\left(i\right)};\lambda \right)\\ \vdots \\ p\left(r^{\left(i\right)}=B|x^{\left(i\right)};\lambda \right) \end{array}\right],\\ =\frac{1}{\sum_{b=1}^{B}e^{\lambda_{b}^{T}x^{\left(i\right)}}}\left[\begin{array}{c} e^{\lambda_{1}^{T}x^{\left(i\right)}}\\ e^{\lambda_{2}^{T}x^{\left(i\right)}}\\ \vdots \\ e^{\lambda_{B}^{T}x^{\left(i\right)}} \end{array}\right], \end{array}$$

where *λ*=[*λ*_1_, *λ*_2_,…,*λ*_*B*_]^*T*^ represents the function coefficients. It can be noted that the softmax function classifier guarantees that the output values are all positive and the sum of them is one. Therefore, the softmax function is able to transform the outputs of the deep neural network to be the predicted probabilities for different concerned classes.

## 3. Proposed Deep Multi-Scale Residual Connected Model

In this study, a novel deep learning-based multi-scale residual connected model is proposed for time-series data processing and athlete performance evaluation. In this section, the proposed method is illustrated in detail, which consists of residual connection, multi-scale feature extraction, and end-to-end relationship model.

### 3.1. Residual Connection

**Figure 1:**
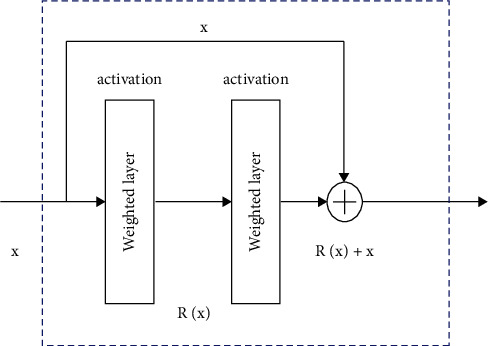
The proposed residual connected scheme in the deep neural network framework.

In the traditional deep neural network, the back-propagation optimization method is usually used for model parameter updates. However, as the model architecture is typically deep with multiple layers, the optimization efficiency is not satisfactory in most cases due to the gradient vanishing problem, which makes the deep neural network difficult to achieve the optimal performance. Therefore in this study, a residual connected neural network scheme is proposed, which is illustrated in Figure 1. The residual connected module generally consists of three main characteristics.

- A short-cut connection is used, which makes the information of the data can propagate through different layers, and directly into the subsequent layers in the network.
- With the residual connected module, deep neural network architecture can be adopted, since the gradient vanishing problem can be largely solved.
- The residual connected module is a relatively independent module with respect to the deep neural network structure, which can be readily added and removed from the existing architecture. Limited additional costs will be introduced for using the residual connected module.

Specifically, the residual connected module can be defined as

(6) $$\begin{array}{c} c=R\left(x,\left\{v_{i}\right\}\right)+x, \end{array}$$

where **x** and **c** denote the input data and output data for the layer, respectively. The function *R* represents the residual connected operation. For example, *R*=**v**_2_*η*(**v**_1_^*T*^**x**) can be used for a simple structure with the weights **v**_*i*_. The practical implementation of the residual connected operation is realized by the short-cut and element-wise sum. The non-linear activation function can be used either before the sum or after the sum.

### 3.2. Multi-Scale Feature Extraction

In this study, a multi-scale feature extraction scheme is proposed to better learn the new features from the raw collected data. Specifically, the filter size in the convolutional operation plays an important role in the automatic feature learning process. Large filter size indicates that the learned features are more general and global with respect to the input data. Correspondingly, smaller filter size means the model pays more attention on the local features. In the current literature, there is no general consensus of the optimal selection of the filter size. Therefore, in this study, we propose to use multiple filter size for the feature extraction, in order to both take advantage of the global and local features from the input data.

**Figure 2:**
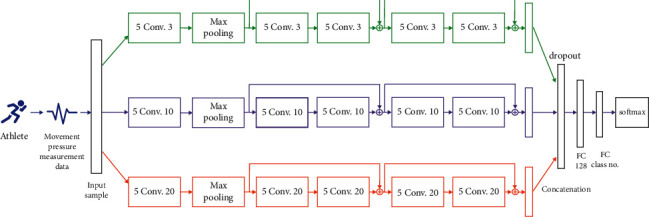
Architecture of the proposed deep multi-scale residual connected neural network model.

In the deep neural network structure, three data and feature streaming approaches are proposed as shown in Figure 2. In each approach, a certain size of the convolutional filter is utilized. The common range of the filter sizes is covered in this study, and they are set as 3, 10, and 20, respectively.

In this way, a single scale of the high-level features is obtained in each approach. After data processing with multiple residual connected blocks, the learned features are concatenated, and further connected with a fully connected layer for information aggregation. Therefore, the final features are in multiple scales and hold richer information from the raw data.

### 3.3. Deep Neural Network Structure

In this study, a deep convolutional neural network structure is used, with the residual modules and the multi-scale feature extraction method. In the proposed framework, the raw measured data are directly used as the input of the deep neural network, which means no prior expertise on the signal processing is required, which largely facilitates the practical utilization of the proposed method in the real scenarios.

Specifically, the neural network architecture is shown in Figure 2. The proposed model consists of multiple residual connected blocks. Each residual connected block typically has two convolutional layers with multiple filters of different sizes. The feature extraction scheme in three scales is generally considered. Correspondingly, three sizes of the convolutional operation are adopted in different feature extraction modules.

After feature extraction of two residual blocks in each module, the learned high-level features of different modules are concatenated for information fusion. Afterward, one fully-connected layer with 128 neurons are used, as well as the final fully-connected layer. Each neuron in the last fully-connected layer represents the predicted confidence value for each class. The softmax function at the end of the structure interprets the confidence values into the probabilities.

In the practical implementations, zero-padding operation in the convolutional layers is adopted to keep the feature map dimension unchanged. The max-pooling is also utilized in the deep model for accelerating the training process and obtaining the significant features. Throughout the deep neural network, the leaky rectified linear unit (Leaky ReLU) activation function is adopted after the layers, which are generally stable with respect to the gradient vanishing or gradient diffusion problems and can lead to better performance. The popular cross-entropy loss function is utilized for optimization of the neural network model [48]. The back-propagation algorithm is applied for the specific changes of the model coefficients in each optimization iteration. The widely used Adam optimization method is employed for model training.

### 3.4. General Implementation

**Figure 3:**
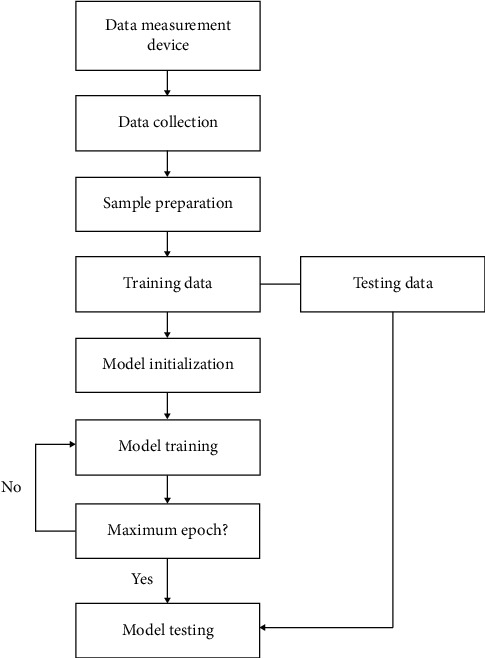
The flowchart of the proposed method in athlete balance control ability evaluation.

Figure 3 shows the flowchart of the proposed deep multi-scale residual connected model. First, the measured time series raw data are prepared into multiple samples. Specifically, in this study, the movement pressure data in two directions are used, i.e., *x* and *y* directions. Therefore, the raw data have two dimensions. The sample dimension in one direction can be defined as *N*_in_, and we can prepare the samples accordingly with dimension [2, *N*_in_]. The raw data can be directly used as the model inputs, and no prior knowledge on signal processing is needed, which shows that the applicability of the proposed method in the real scenarios is strong.

Next, with respect to the specific dataset information, the proposed deep multi-scale residual connected neural network architecture is established, and the detailed configurations are determined, including the number of neurons in the hidden layers, number of convolutional filters, and so on. In order to start the model training process, the data samples are fed into the network. Through multiple layers of feature extraction, high-level representations are obtained, which are used for the final classification. Back-propagation algorithm is used for the updates of the model parameters.

Afterward, when the model training process is finished, the testing samples are fed into the deep neural network to test the model performance with respect to the unseen data.

## 4. Experimental Study

### 4.1. Dataset and Task Description

In this study, a real athlete balance control ability evaluation dataset is used for the validation of the proposed method. Specifically, multiple freestyle skiing aerials athletes of different balance control levels are asked to stand still on a balance meter under feet. The area of the balance meter is 65 cm × 40 cm, and the balance meter can collect the movement pressure data in the anteroposterior and mediolateral directions.

**Figure 4:**
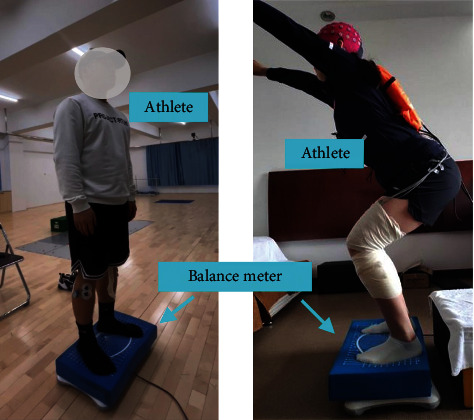
The scenarios of the freestyle skiing aerials athlete movement pressure data collection experiments.

Three levels in balance control of freestyle skiing aerials athletes are considered, which are denoted as high-level (H), medium-level (M), and normal people (N), respectively. Each level includes two athletes, who are represented by numbers of #1 and #2, respectively. The athletes are required to keep balance at their best when they are standing on the balance meter. Their upper bodies are supposed to be stationary, and the noise of the environment is kept at the minimum level. The athletes use two of their feet for standing with their eyes closed to focus on the data measurement. The movement pressure data sampling frequency is 100 Hz.  Table 1 presents the information of the dataset used in this study, and Figure 4 shows the scenario of the experiment.

In this study, different athlete balance control ability prediction tasks are considered in order to fully examine the performance of the proposed method. Specifically, with respect to the dataset, four tasks are implemented, where different training and testing data are used. The tasks are demonstrated in Table 2. Different athletes in each levels are used for validation, which cover a wide range of the experimental settings and provide fair evaluations of the performance of the proposed method.

### 4.2. Model Establishment

In this study, mini-batch data samples are used to implement the stochastic gradient descent (SGD) optimization method for updating the deep neural network parameters. In each epoch of the training process, the training data samples are divided into different mini-batches in a random manner. Eight samples are included in each mini-batch with the corresponding label information.

Afterward, the deep neural network parameters are updated with the popular cross-entropy loss function with respect to each mini-batch. It is worth noting that the dimension of the data samples plays an important role in the model performance. Larger dimension indicates more information is included in each sample. However, higher computational burden usually exists. Therefore, this is generally a trade-off in the practical applications.

The deep neural network model architecture is shown in Figure 2. The model performance can be affected by some key factors, such as the convolutional filter size and number. Those will be further investigated in the following sections in this study. Specifically, for the experiments, the parameters used in the proposed method are listed in Table 3, which are selected based on the performances on the validation data in this case.

### 4.3. Compared Approaches

The proposed deep multi-scale residual connected neural network model offers a new perspective for big data-driven intelligent athlete balance control performance evaluation. In this study, similar methods in the existing literature are also implemented for comparisons, in order to examine the effectiveness and superiority of the proposed methodology. Specifically, the following approaches are considered, which cover a wide range of popular techniques for data-driven studies.

#### 4.3.1. NN

The basic neural network (NN) model is firstly considered, which follows a typical pattern for neuron connections [23]. Specifically, a multi-layer perceptron structure is used, which includes one hidden layer with 1000 neurons. Similar configurations are used as the proposed method, such as the Leaky ReLU activation function and dropout operation.

#### 4.3.2. DNN

The deep neural network (DNN) is an extension of the basic neural network structure [49]. Three hidden layers are considered in the DNN method in this study, which consists of 1000, 1000, and 500 neurons, respectively. Similarly, the Leaky ReLU activation function is also employed, as well as the dropout technique.

#### 4.3.3. DSCNN

The deep single-scale convolutional neural network (DSCNN) method is implemented [50], which share the similar architecture with the proposed method, except for the multi-scale feature extraction scheme. Specifically, only one data processing approach is considered in the network. Correspondingly, one convolutional filter size is employed for the feature extraction. No feature concatenation is used at the fully-connected layers. The other settings are similar with the proposed method.

#### 4.3.4. WORes

The WORes method represents the deep multi-scale convolutional neural network architecture, which does not have the residual connected schemes [40]. Specifically, the short cut connections between the convolutional layers are removed from the proposed method. This approach is a comparison to show the benefits of the proposed residual connected scheme.

With respect to all the compared methods in this study, the cross-entropy loss function is used for classification of the athlete balance control performance. The Adam optimization method is adopted for the model updates with the mini-batch data sample selections. The same learning rate is used as the proposed method.

## 5. Experimental Results and Performance Analysis

In this section, the experimental results of the proposed method on different athlete balance control ability evaluation tasks are presented, as well as the results of different compared methods. Ablation studies are also extensively carried out to evaluate the influence of different key parameters of the proposed method on the model performance. In order to provide fair results and comparisons, each experiment is implemented for three times, and average results are presented.

**Figure 5:**
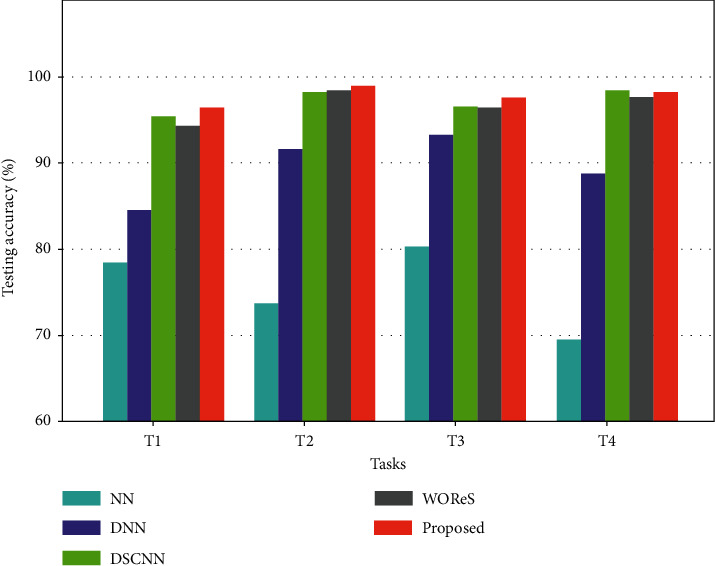
The experimental results of different compared methods in different athlete balance control ability evaluation tasks.

Figure 5 shows the general experimental results using different methods in different tasks. It can be observed that in general, the neural network-based methods are able to achieve good evaluation results, and the testing accuracies are high. The testing accuracies of the basic NN method are not competitive in different tasks, and less than 80% accuracies are obtained. This indicates that the shallow network structure cannot well capture the underlying pattern of the massive data. The DNN method achieves significantly higher testing accuracies in different tasks, and the accuracies are basically higher than 90%. The results show that the deep architecture can well learn the highly nonlinear relationship between the movement pressure measurement data and the athlete balance control ability. The DSCNN and WORes methods are quite competitive in this problem, and the testing accuracies in different cases are mostly higher than 95%. However, the optimal performance is generally achieved by the proposed deep multi-scale residual connected model. Close to 100% testing accuracies in different tasks can be obtained. Noticeable improvements can be observed compared with the DSCNN and WORes methods. This implies that the proposed multi-scale feature learning scheme and residual connected structure can well enhance the learning performance of the deep neural network architecture, and they are well suited for the athlete balance control ability evaluation problem by processing the time-series pressure data.

### 5.1. Effect of Convolutional Filter Number

**Figure 6:**
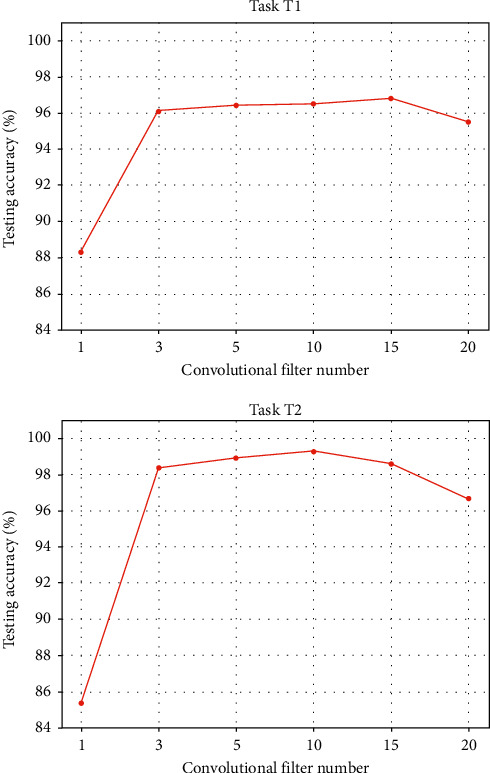
The influence of the number of convolutional filters on the model performance in the tasks T1 and T2.

The number of convolutional filters in the layers throughout the deep neural network plays an important role in affecting the model performance. Fewer convolutional filters are generally less effective in learning the complicated patterns from the data, and more convolutional filters will basically lead to better performance with enhanced learning capacity. However, the overfitting issue may occur since larger model architecture and more parameters are included. In this section, this issue is investigated, and the effects of the convolutional filter number on the model performance are presented in Figure 6. The tasks T1 and T2 are used for investigation.

It can be observed that in general, the influence of the convolutional filter number on the testing accuracies is not quite significant when the number is not very small. When only one convolutional filter is used, remarkably low testing accuracies are obtained, which are lower than 90%. However, when more convolutional filters are applied, the testing accuracies are generally stable and higher than 95%. When 20 convolutional filters are employed, slight performance drops are observed. Nonetheless, this does not have noticeable influence on the general model performance. Therefore, when the number is not too small with a reasonable value, promising results can be basically achieved.

### 5.2. Effect of Sample Number

**Figure 7:**
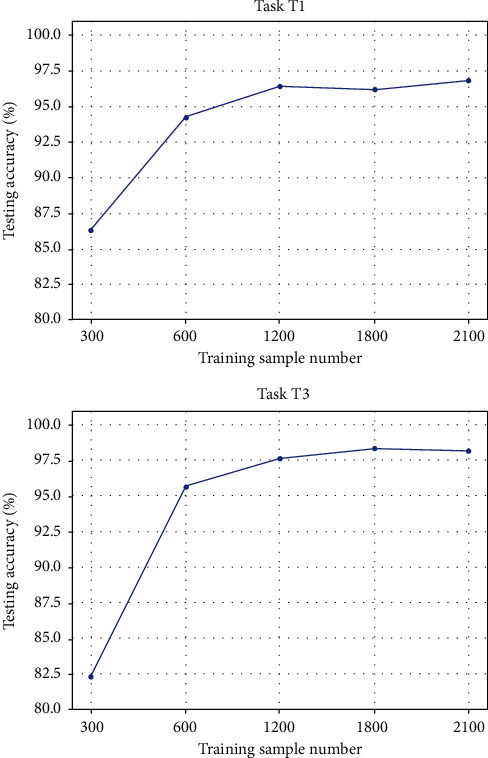
The influence of the sample number on the model performance in the tasks T1 and T3.

In this section, the effects of the sample number on the model performance are investigated. The number of training samples is also an important parameter in the data-driven methods. Generally, more training samples lead to better performances. However, since the data are usually expensive in different areas, it is always preferred to achieve good performance with minimum data. The experimental results are presented in Figure 7. The tasks T1 and T3 are focused on in this section.

It is noted that the experimental results are basically in accordance with our understanding in the literature. When 300 training samples are used, lower testing accuracies are obtained in different tasks, which are lower than 87%. When more training samples are employed, the results significantly become better and higher than 92% testing accuracies are basically obtained. When the sample number is larger than 600, small fluctuations of the testing performance are observed. However, the performances are generally stable with respect to different sample numbers. It is also noted that 600 training samples are mostly sufficient for building the deep neural network model for this problem, which can be considered as the minimum number that the model requires.

### 5.3. Visualization of Learned Representation

**Figure 8:**
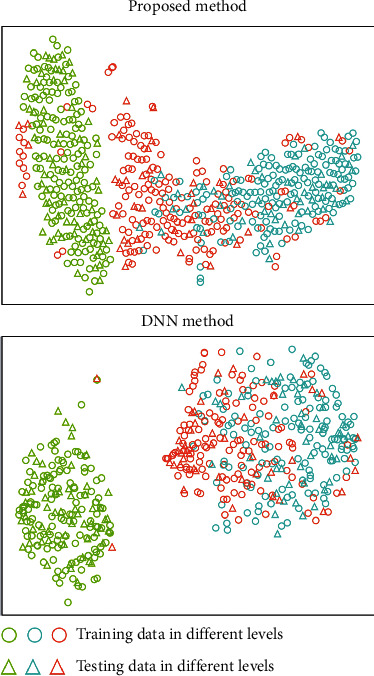
The visualization results of the learned features by different methods in task T1.

**Figure 9:**
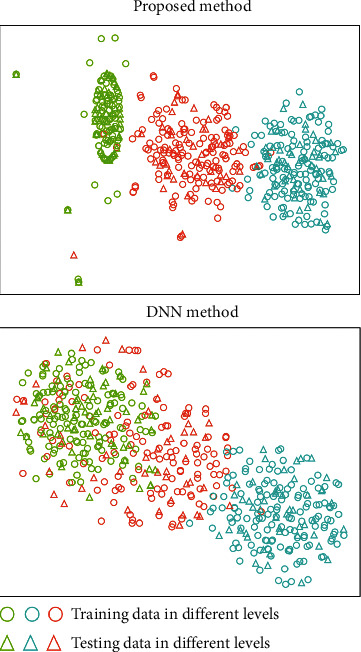
The visualization results of the learned features by different methods in task T4.

In this section, the learned features by the deep neural network models are visualized to show the effectiveness of the methods. Specifically, the high-level representations of the samples at the last fully-connected layer are considered. The t-SNE method is adopted for dimension reduction of the learned high-dimensional features. Two new dimension can be obtained and plotted for visualizations. The results in the tasks T1 and T4 are shown in Figures 8 and 9 respectively.

It can be observed that using the proposed deep multi-scale residual connected neural network method, different classes are more separated with respect to the learned features. Limited overlappings between different classes are found, which validates that the proposed method can achieve high testing accuracies for the classification tasks. The DNN method is less competitive in the cases. Noticeable overlappings between different classes are observed in the learned feature sub-space, and some data samples are also located outside the clusters of their own classes. This shows that the DNN method is less effective than the proposed method in the tasks. It should be pointed out that the NN method is far less effective in the case studies, and the visualization results do not carry sufficient information for demonstrating the effects. The results in this section validate the effectiveness of the proposed method in an intuitive way, which shows that the proposed method is quite promising for automatic athlete balance control ability evaluation.

## 6. Conclusion

In this study, a deep multi-scale residual connected neural network model for intelligent athlete balance control ability evaluation. The time-series pressure measurement data under-feet are processed and analyzed. The raw data are directly used as the model input for automatic evaluations. No prior knowledge on signal processing is needed, which makes it easy for real applications. A multi-scale feature extraction scheme is proposed, which utilize the learned features from different types of convolutional filters. The information fusion of the learned features further enhances the model training ability. The proposed residual connected blocks can effectively increase the model training efficiency while keeping the training quality. This is well suited for the deep neural network architecture and can be readily applied in different network structures. Experiments on the real athlete under-feet pressure measurement data are carried out for validations. The results show that the proposed method is promising for intelligent evaluations of the athlete balance control abilities, and offers a new perspective in mining athlete measurement data.

The advantage of the proposed method lies in the end-to-end modeling structure, which makes the balance control ability evaluation task more straight-forward to implement. On the other hand, despite the promising results, it should be pointed out that main drawback of the proposed method lies in the structure of the neural network model, since three network approaches are considered in the model, which is a little complex for the data-driven model. Further research works will be carried out on the optimization of the deep neural network architecture while retaining the model performance.

## Figures and Tables

**Table 1:** Information of the athlete movement pressure measurement dataset used in this study.

Athlete level	No. of athletes	Code names	Sampling frequency

H (High-level athlete)	2	H#1, H#2	100 Hz
M (Medium-level athlete)	2	M#1, M#2	100 Hz
N (Normal people)	2	N#1, N#2	100 Hz

**Table 2:** Information of different athlete balance control ability evaluation tasks used in this study.

Task name	Concerned	No. of training	No. of testing
	Athletes	Samples	Samples
T1	H#1, M#1, N#1	1200	600
T2	H#2, M#2, N#2	1200	600
T3	H#1, M#2, N#1	1200	600
T4	H#2, M#1, N#2	1200	600

**Table 3:** Parameters of the proposed method used in this study.

Parameter	Value	Parameter	Value

Batch size	8	Sample dimension	200 *∗* 2
Epoch number	100	Convolutional filter size	3, 10, 20
Learning rate	1*∗*10^−4^		

## Data Availability

The data used to support the findings of this study are included within the article.
